# Long intergenic non-protein coding RNA 662 accelerates the progression of gastric cancer through up-regulating centrosomal protein 55 by sponging microRNA-195-5p

**DOI:** 10.1080/21655979.2021.2023978

**Published:** 2022-01-17

**Authors:** Fei Tao, Likun Qi, Guoqing Liu

**Affiliations:** a Department of Oncology, Qinghai Provincial People’s Hospital, Xining, China; b Department of Gastrointestinal Surgery, Fifth People’s Hospital of Qinghai Province, Xining, China

**Keywords:** Gastric cancer, LINC00662, miR-195-5p, CEP55

## Abstract

Long non-coding RNAs (lncRNAs) are important players in regulating diverse human diseases, including cancers. Nonetheless, the function of long intergenic non-protein coding RNA 662 (LINC00662) in gastric cancer (GC) carcinogenesis and progression remains to be delineated. In the present study, LINC00662, microRNA-195-5p (miR-195-5p) and centrosomal protein 55 (CEP55) mRNA expression levels were quantified by qRT-PCR. GC cell proliferation, migration and invasion were analyzed by CCK-8, BrdU and Transwell assays. Besides, dual-luciferase reporter and RNA pull-down assays were conducted for verifying the targeting relationships of LINC00662, miR-195-5p and CEP55. The regulatory functions of LINC00662 and miR-195-5p on CEP55 were examined utilizing Western blot. In this study, it was revealed that LINC00662 expression level was elevated in GC tissues and cells. LINC00662 overexpression facilitated the malignant biological behaviors of GC cells whereas knockdown of LINC00662 worked oppositely. In terms of mechanism, LINC00662 targeted miR-195-5p to modulate CEP55 expression. In conclusion, LINC00662 facilitates the malignant biological behaviors of GC cells via miR-195-5p/CEP55 axis, and therefore, it may be a promising target for GC treatment.

## Introduction

Gastric cancer (GC) is a life-threatening cancer, which is with high incidence in East Asia [1,2]. A majority of patients unfortunately were in the advanced stage by the time of diagnosis, frequently accompanied by lymph node or distant metastasis [3]. Delineating the molecular mechanism of GC progression is vital.

Long non-coding RNAs (lncRNAs), incapable of encoding proteins, is able to interact with DNA, RNA or proteins, and they can regulate gene expression via multiple mechanisms [4,5]. In recent years, it has been elucidated that lncRNAs are associated with tumorigenesis [6–8]. Long intergenic non-protein coding RNA 662 (LINC00662), an emerging lncRNA identified in recent years, reportedly, is associated with the progression of oral squamous cell carcinoma, hepatocellular carcinoma and lung cancer [9–11]. Intriguingly, it is reported that silencing LINC00662 impedes GC cell proliferation and increases chemosensitivity [12]. Nonetheless, the detailed biological functions and underlying mechanism of LINC00662 in GC progression require further delineation.

MicroRNAs (miRNAs) take part in regulating a lot of fundamental biological processes [13,14]. MiR-195-5p regulates disease progression and is abnormally expressed in some types of cancers [15–17]. Importantly, miR-195-5p overexpression suppresses YAP-mediated Wnt/β-catenin signaling pathway and represses GC progression [17].

Centrosomal protein 55 (CEP55), as a regulator in spindle assembly, exhibits regulatory functions in cytokinesis [18]. CEP55, overexpressed in glioma tissues and cells, facilitates cancer cell proliferation and suppresses apoptosis through PI3K/Akt/p21 signaling pathway [19]. CEP55 also facilitates epithelial-mesenchymal transition of renal cell carcinoma cells via PI3K/AKT/mTOR pathway [20]. The abnormal high expression of CEP55 also contributes to GC progression [21]. Nevertheless, the upstream regulatory mechanism of CEP55 in GC warrants further investigation.

This work was aimed to decipher how LINC00662 functioned in GC progression. It was hypothesized that, LINC00662 could promote GC progression as a competitive endogenous RNA (ceRNA). It was revealed that LINC00662 was highly expressed in GC, and it, via repressing miR-195-5p and up-regulating CEP55, increased the malignancy of GC cells.

## Materials and methods

### Samples collection

From March 2018 to March 2019, GC tissues and adjacent non-tumor tissues obtained during surgery in Qinghai Provincial People’s Hospital were rapidly frozen in liquid nitrogen. All of the subjects signed the informed consent. The inclusion criteria: patients with gastric adenocarcinoma receiving no neoadjuvant therapy. Exclusion criteria: adenocarcinoma of esophagogastric junction; patients receiving neoadjuvant therapy; patients with severe cardiovascular disease, cerebrovascular disease, metabolic diseases and other cancers; patients with incomplete clinicopathologic information. This work was permitted by the Ethics Committee of Qinghai Provincial People’s Hospital (Approval No. 20,170,334). This collection and usage of human samples followed Declaration of Helsinki.

### Cell culture and transfection

American Type Culture Collection (ATCC, Manassas, VA, USA), together with China Center for Type Culture Collection (CCTCC, Wuhan, China), provided GC cell lines (SGC-7901, MKN28, BGC823 and MGC-803), immortalized gastric epithelial cell line (GES-1) and kidney epithelial cell line HEK293 cells. In DMEM (Hyclone, Logan, UT, USA) supplemented with 10% FBS (Hyclone, Logan, UT, USA), 100 U/mL penicillin and 100 μg/mL streptomycin (Hyclone, Logan, UT, USA), these cells were accordingly cultivated at 37°C in 5% CO_2_.

LINC00662 and CEP55 overexpression vectors, pcDNA3.1 empty vector (Thermo Fisher Scientific, Wilmington, DE, USA), siRNAs targeting LINC00662 (si-LINC00662), siRNA targeting CEP55 (si-CEP55) and siRNA negative control (si-NC), miR-195-5p mimic (miR-195-5p), miRNA negative control (miR-con), miR-195-5p inhibitor (miR-195-5p in) and inhibitor negative control (miR-in) (GenePharm, Shanghai, China) were transfected into the cells using Lipofectamine^TM^ 2000 Reagent (Invitrogen, Waltham, MA, USA).

### Quantitative real-time polymerase chain reaction (qRT-PCR)

TRIzol reagent (Invitrogen, Carlsbad, CA, USA) was utilized to conduct RNA extraction. mRNA and miRNA were reversely transcribed employing the Transcriptor First Strand cDNA Synthesis kit (Roche, Basel, Switzerland), and the miRNA 1st Strand cDNA Synthesis Kit (by stem-loop) (NovaBio, Shanghai, China), respectively. A SYBR® Premix DimerEraser Kit (TaKaRa, Dalian, China) was utilized to conduct qRT-PCR on Applied Biosystems 7500 Real-Time PCR System, with GAPDH and U6 as the internal references. Below were the primers: LINC00662, 5ʹ-CGGGCGATTATCGACGATC-3ʹ (forward), and 5ʹ-TCGGGATCGACTACCCTAGGTAC-3ʹ (reverse); miR-195-5p, 5ʹ-GGGGTAGCAGCACAGAAAT-3ʹ (forward), and 5ʹ-TCCAGTGCGTGTCGTGGA-3ʹ (reverse); CEP55, 5ʹ-TTGGAACAACAGATGCAGGC-3ʹ (forward), and 5ʹ- GAGTGCAGCAGTGGGACTTT-3ʹ (reverse); GAPDH, 5ʹ-TGTTCGTCATGGGTGTGAAC-3ʹ (forward), and 5ʹ-ATGGCATGGACTGTGGTCAT-3ʹ (reverse); U6, 5ʹ-CGGGTGCTCGCTTCGCAGC-3ʹ (forward), and 5ʹ-CCA GTGCAGGGTCCGAGGT-3ʹ(reverse).

### Subcellular fractionation

A Cytoplasmic and nuclear RNA purification kit (Amyjet, Wuhan, China) was adopted to extract cytoplasmic and nuclear RNA from SGC-7901 and BGC823 cells.

### Cell counting kit-8 (CCK-8) assay

The transfected GC cells were inoculated into 96-well plate (1000 cells per well), and cultured. After 12, 24, 48, 72 and 96 h, 10 μL of CCK-8 reagent (Dojindo, Kumamoto, Japan), was added into each well to incubate BGC823 and SGC-7901 cells for 2 h. Subsequently, OD_450nm_ was determined with a microplate reader.

### Bromodeoxyuridine (BrdU) staining

The transfected GC cells in each group were transferred onto coverslips (Beyotime, Shanghai, China) and then cultured for 12 h. Subsequently, the cells were incubated BrdU solution (Beyotime, Shanghai, China) for 6 h. The GC cells, after the medium was discarded, were fixed with 4% paraformaldehyde for 30 min, and incubated with anti-BrdU antibody (Beyotime, Shanghai, China) at ambient temperature for 1 h, and then rinsed with phosphate buffer saline (PBS). Subsequently, the nuclei were stained employing Hoechest staining solution (Beyotime, Shanghai, China). After the cells were washed by PBS again, the numbers of BrdU and Hoechst positive cells were counted.

### Transwell assays

After transfection, BGC823 and SGC-7901 cell lines (1 × 10^5^ cells/ml) were harvested for the preparation of single-cell suspensions with serum-free medium. Then, the upper and lower compartments of each Transwell chamber (Corning, Shanghai, China) were added with 200 μL of single-cell suspension and medium supplemented with 20% FBS, respectively. After 36 h, the cells upper surface of the membrane were removed, and the cells on the below surface of the membrane were fixed with 4% paraformaldehyde and then stained with 0.5% crystal violet solution. Finally, the stained cells were observed and counted under a microscope. Notably, the membranes, prior to usage in the invasion assay, were coated with a layer of Matrigel (5.00 μg/well; BD, San Jose, CA, USA).

### Xenograft model

Animal experiment was authorized by the Animal Protection and Use Committee of Qinghai Provincial People’s Hospital (Approval No. 20,200,085). Female BALB/c nude mice available from SIPPR-BK Laboratory Animal Co. Ltd. (Shanghai, China) were divided into NC group and LINC00662 overexpression group, 15 mice in each group. BGC823/NC and BGC823/LINC00662 cells were injected into the nude mice via caudal vein (1 × 10^7^ per mouse). The mice, after 2 weeks, were euthanized, and the lung metastases were checked with hematoxylin-eosin (HE) staining by the pathologists.

### Dual-luciferase reporter assay

PmiR-RB-REPORT™ (Ribobio, Guangzhou, China) reporter plasmids containing the wild-type LINC00662 and CEP55 3ʹ-UTR sequences with predicted binding sites for miR-195-5p were constructed. HEK293 cells were co-transfected with the reporter plasmids and miR-195-5p mimics or miR-con, and after 48 h, the cells were collected to examine the luciferase activities.

### RNA pull-down assay

Biotin-labeled miR-195-5p (Bio-miR-195-5p), miR-con (Bio-miR-con), LINC00662 (Bio-LINC00662) and negative control (Bio-NC) were provided by Sangon Biotech (Shanghai, China). Briefly, transfected with the above probes, SGC-7901 and BGC823 cells were cultured for 2 d and lysed, followed by the incubation with magnetic beads at 4°C for 3 h. Finally, the relative enrichment of LINC00662 or miR-195-5p in the complex was detected.

### Western blot

RIPA lysis buffer (Beyotime, Shanghai, China) was adopted to conduct protein extraction, with the concentration of protein samples determined with a Bicinchoninic acid (BCA) kit (Beyotime, Shanghai, China). After SDS-PAGE, the proteins on the gel were transferred onto PVDF membranes (Beyotime, Shanghai, China). After that, the membranes were incubated with anti-CEP55 rabbit polyclonal antibody (ab170414, 1:1000, Abcam, Shanghai, China) and anti-GAPDH rabbit polyclonal antibody (ab181602, 1:2000, Abcam, Shanghai, China) overnight at 4°C, followed by the rinse with tris buffered saline tween (TBST) 3 times, with 15 min each time. Following that, the membranes were incubated, at ambient temperature, with horseradish peroxidase (HRP)-labeled secondary antibodies (1: 2000, Beyotime, Shanghai, China) for 1 h and subsequently, washed with TBST with the same procedure. After adding the enhanced chemiluminescence kit (Beyotime, Shanghai, China) onto the membranes, the protein bands were detected employing ChemiDoc™ Touch Imaging System (Bio-Rad, Shanghai, China).

### Bioinformatics analysis

The targeting relationships among LINC00662, miR-195-5p and CEP55 were predicted by StarBase database (http://starbase.sysu.edu.cn/) [22].

### Statistical analysis

SPSS 22.0 statistical software (SPSS Inc., Chicago, IL, USA) was employed to analyze the data, which were shown as ‘mean ± standard deviation’. The difference between two groups was compared employing student’s *t*-test. Pearson’s correlation analysis was used to evaluate the correlation. *P* < 0.05 implicated statistical significanc.

## Result

The present work explored the expression levels of LINC00662 and miR-195-5p in GC, and investigate their biological function in modulating the malignant phenotypes of GC cells with *in vitro* and *in vivo* experiments. Additionally, the regulatory functions of LINC00662 and miR-195-5p on CEP55 were studied. Our study showed that LINC00662, miR-195-5p and CEP55 formed a ceRNA network to regulate the progression of GC.

### LINC00662 and miR-195-5p in GC tissues and cells

**Figure 1.**
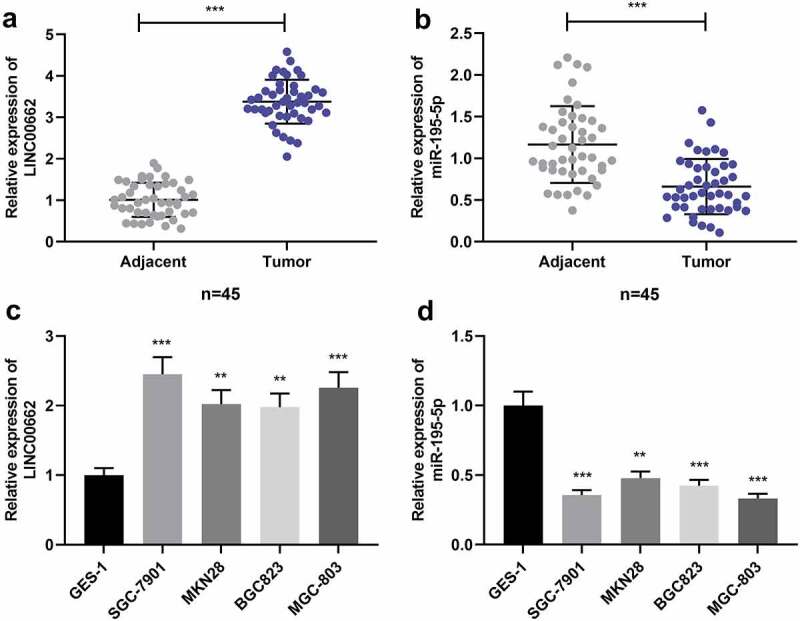
LINC00662 was up-regulated in GC but miR-195-5p was decreased.

First of all, the expression levels of LINC00662 and miR-195-5p in GC tissues and cell lines were detected by qRT-PCR. It was revealed that, LINC00662 was markedly elevated while miR-195-5p expression was observably decreased in GC tissue samples (Figure 1(a,b)). Additionally, LINC00662 was uncovered to be highly expressed whereas miR-195-5p was under-expressed in GC cells (Figure 1(c,d)).

a-b. LINC00662 expression and miR-195-5p expression in GC tissues samples (N = 45) and non-cancerous tissue samples (N = 45) were quantified by qRT-PCR. C-D. qRT-PCR was adopted for quantifying LINC00662 expression (c) and miR-195-5p expression (d) in different cell lines. All of the experiments were performed in triplicate. **P < 0.01 and *** *P* < 0.001

### LINC00662 was a molecular sponge of miR-195-5p

**Figure 2.**
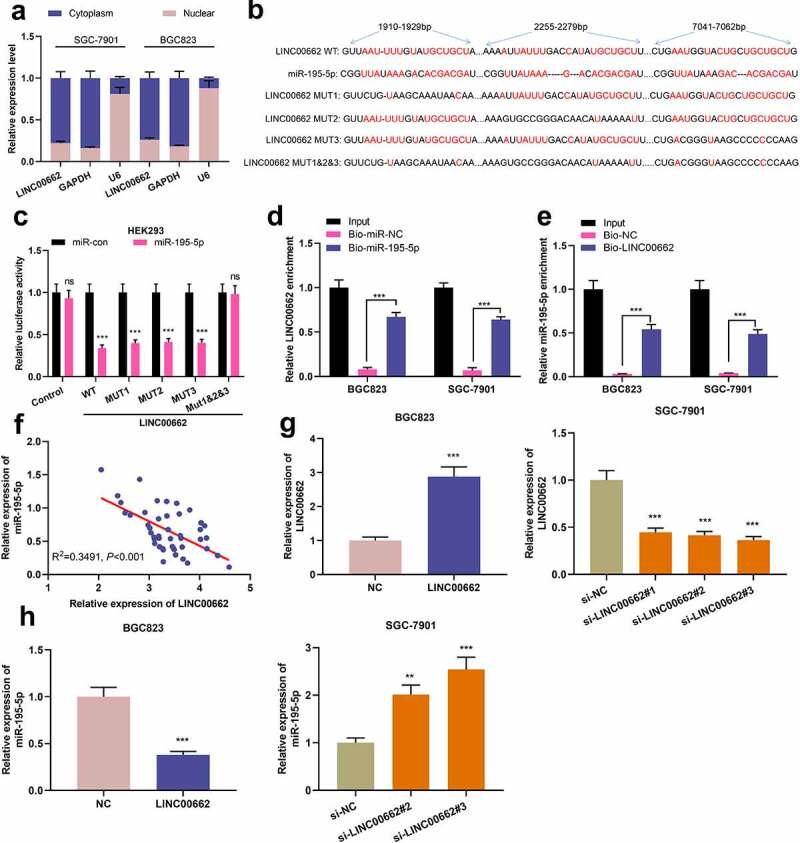
MiR-195-5p was the target of LINC00662.

LINC00662 was mainly located in the cytoplasm of BGC823 and SGC-7901 cells, showing that it probably modulated miRNA as a ceRNA (Figure 2(a)). StarBase database (http://starbase.sysu.edu.cn/) indicated 3 potential complementary binding sites between miR-195-5p and LINC00662 (Figure 2(b)). As shown, miR-195-5p overexpression repressed the luciferase activity of LINC00662-WT, LINC00662-MUT1, LINC00662-MUT2 and LINC00662-MUT3 vectors but had no obvious effect on LINC00662-MUT1&2&3 vector (Figure 2(c)). Moreover, compared with Bio-miR-NC, LINC00662 could be enriched by Bio-miR-195-5p probe (Figure 2(d)); compared with Bio-NC, miR-195-5p was observably enriched in the RNA complex pulled down by bio-LINC00662 probe (Figure 2(e)). Additionally, LINC00662 expression and miR-195-5p expression exhibited a negative correlation in GC tissues (Figure 2(f)). Furthermore, LINC00662 overexpression plasmid was transiently transfected into BGC823 cells, which were with the lowest expression of LINC00662, while LINC00662 siRNAs (si-LINC00662#1, si-LINC00662#2, si-LINC00662#3) were transfected into SGC-7901 cells which were with the highest expression of LINC00662 (Figure 2(g)). si-LINC00662#3 was, due to its high knock-down efficiency, selected for the following experiments. Subsequently, it was revealed that miR-195-5p expression was decreased in BGC823 cells overexpressing LINC00662, whereas miR-195-5p expression was promoted in SGC-7901 cells with LINC00662 silenced, implying that miR-195-5p was a direct target of LINC00662 and negatively regulated by LINC00662 (Figure 2(h)).

a. qRT-PCR was used to detect LINC00662 expression in nuclear and cytoplasmic fractions of BGC823 and SGC-7901 cells. B. The binding sequences between miR-195-5p and LINC00662 were predicted. c. Luciferase activities of the cells were detected to validate the predicted binding sites. d-e. RNA pull-down assay was adopted to validate the interaction between miR-195-5p and LINC00662. F. qRT-PCR data were utilized to examine the correlation between miR-195-5p expression and LINC00662 expression in GC samples (N = 45). g. LINC00662 overexpression plasmids were transfected into BGC823 cells, and siRNAs were transfected into SGC-7901 cells, and then the expression of LINC00662 in GC cells was detected by qRT-PCR. h. qRT-PCR was utilized to quantify miR-195-5p expression in GC cell lines after transfection. All of the experiments were performed in triplicate. **P < 0.01, ***P < 0.001 and ns: *P* > 0.05.

### LINC00662 targeted miR-195-5p to facilitate the malignant phenotypes of GC cells

**Figure 3.**
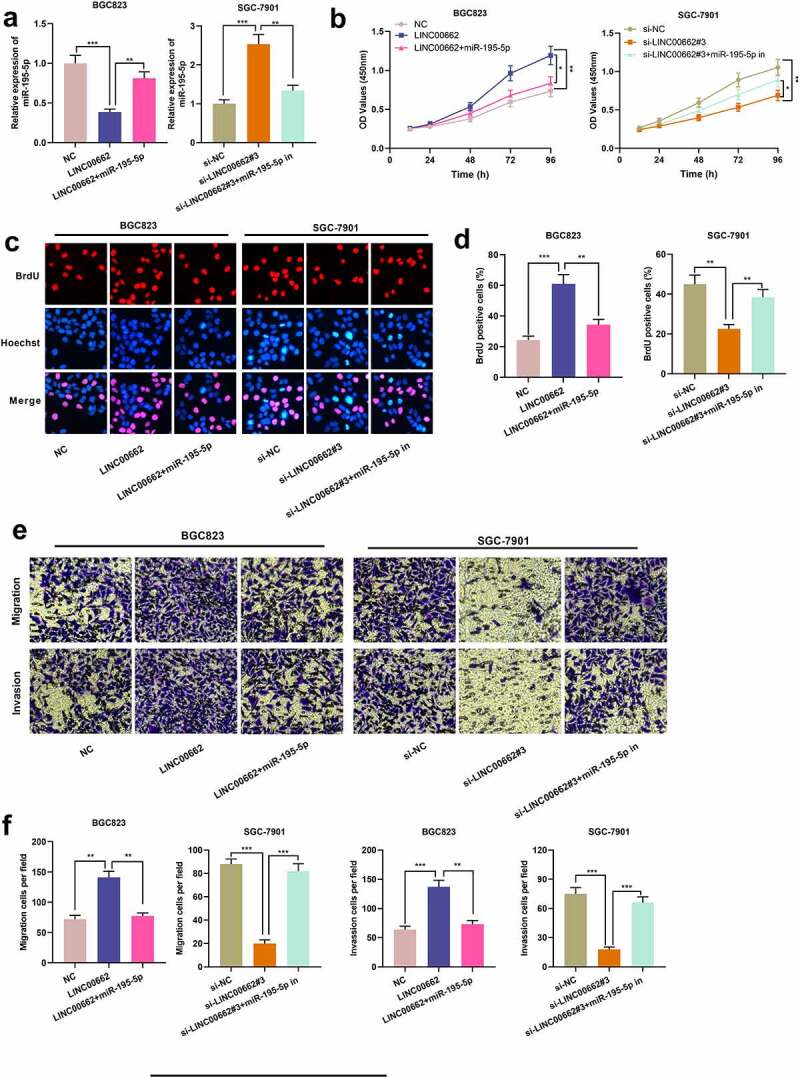
MiR-195-5p could abolish the effects of LINC00662 on biological behaviors of GC cells.

For deciphering how LINC00662/miR-195-5p axis worked in GC progression, BGC823 cells were transfected with LINC00662 overexpression plasmid or co-transfected with miR-195-5p mimics while SGC-7901 cells were transfected with si-LINC00662#3 or co-transfected with miR-195-5p inhibitors (Figure 3(a)). As shown, LINC00662 overexpression promoted the malignant phenotypes of BGC823 cells, whereas knockdown of LINC00662 worked oppositely on SGC-7901 cells (Figure 3(b-f)). Besides, transfecting miR-195-5p mimics weakened the promoting impacts of LINC00662 overexpression on the malignant biological behaviors of GC cells, whereas the inhibitory impacts of si-LINC00662#3 were abolished by miR-195-5p inhibitors (Figure 3(b-f)). Besides, the number of lung metastases in mice injected with BGC823 cells with LINC00662 overexpression was significantly increased; 10 of 15 mice in the BGC823/LINC00662 group showed severe lung metastasis, and the incidence was observably higher than that of BGC823/NC group (2 of 15 mice) (Supplementary Figure S1). Collectively, it was concluded that LINC00662 facilitated the malignant phenotypes of GC cells, probably via sponging miR-195-5p.

a. BGC823 cells were transfected with LINC00662 overexpression plasmid or co-transfected with miR-195-5p mimics, respectively. SGC-7901 cells were transfected with si-LINC00662#3 or co-transfected with miR-195-5p inhibitor, and miR-195-5p expression in cells was quantified by qRT-PCR. b-d. The proliferation of transfected GC cells was evaluated by CCK-8 assay (B) and BrdU assay (c, d). e-f. Transwell experiment was adopted for assessing the migration and invasion of transfected GC cells. All of the experiments were performed in triplicate. *P < 0.05, **P < 0.01 and ***P < 0.001.

### CEP55 was the target of miR-195-5p

**Figure 4.**
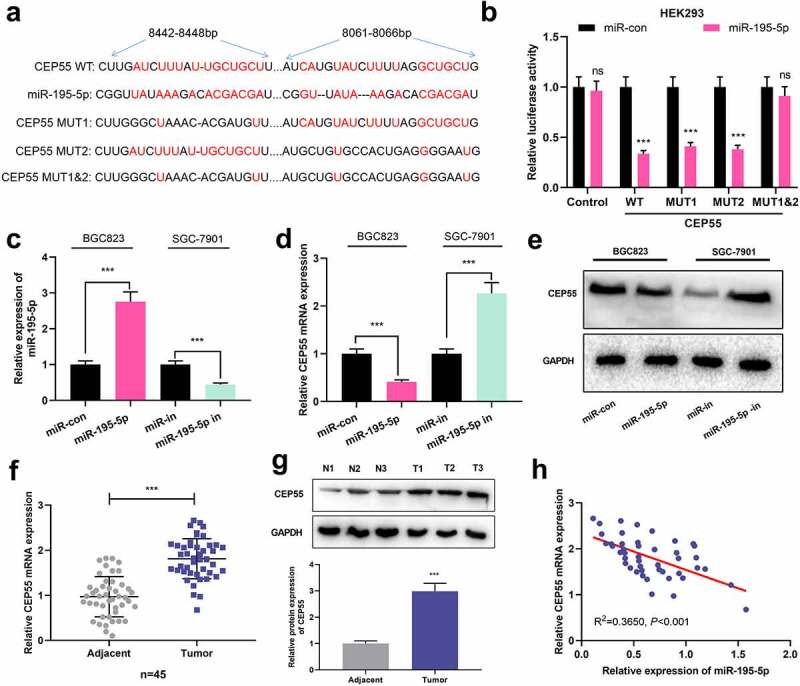
CEP55 was the direct target of miR-195-5p.

For clarifying how miR-195-5p worked in GC, StarBase database (http://starbase.sysu.edu.cn/) was searched again, and it predicted miR-195-5p targeted CEP55 (Figure 4(a)). Subsequently, in dual-luciferase reporter assay, it was revealed that the transfection of miR-195-5p mimics suppressed the luciferase activities of WT, MUT1 and MUT2 CEP55 reporters, whereas no effects were observed on that of MUT1&2 CEP55 reporter (Figure 4(b)). Besides, miR-195-5p in BCG823 cells was elevated after the transfection of miR-195-5p mimics, whereas miR-195-5p in SGC-7901 cells was up-regulated after the transfection of miR-195-5p inhibitors (Figure 4(c)). Moreover, miR-195-5p overexpression repressed the expression of CEP55 mRNA and protein in BGC823 cells but inhibiting miR-195-5p induced the expression of CEP55 mRNA and protein in SGC-7901 cells (Figure 4(d,e)). Additionally, as opposed to normal tissues adjacent to cancer, CEP55 mRNA and protein exhibited enhanced expression in GC tissues (Figure 4(f,g)). Besides, miR-195-5p expression was in negative association with CEP55 mRNA expression in GC tissue samples (Figure 4(h)). These data suggested that CEP55 was a target gene of miR-195-5p and could be negatively modulated by it.

a. Bioinformatics analysis was employed for predicting the binding sequences between miR-195-5p and 3ʹUTR of CEP55. B. Luciferase activity was performed to validate the predicted binding sites after reporter vectors and miR-195-5p mimics (or miR-con) were co-transfected into the cells. c. qRT-PCR was adopted to quantify miR-195-5p in GC cells after the cells were transfected with miR-195-5p mimics or inhibitors. d-e. CEP55 mRNA and protein in GC cells with miR-195-5p overexpressed or inhibited were quantified by qRT-PCR (d) and Western blot (e). F. qRT-PCR was utilized to detect the expression level of CEP55 mRNA in GC tissues (N = 45) and non-cancerous tissues (N = 45). g. Western blot was adopted to quantify CEP55 protein in GC tissues (N = 3) and non-cancerous tissues (N = 3). h. The association between miR-195-5p and CEP55 mRNA expressions in GC samples (N = 45) was evaluated. All of the experiments were performed in triplicate. ***P < 0.01 and ns: *P* > 0.05.

### LINC00662 accelerated GC progression by regulating CEP55

**Figure 5.**
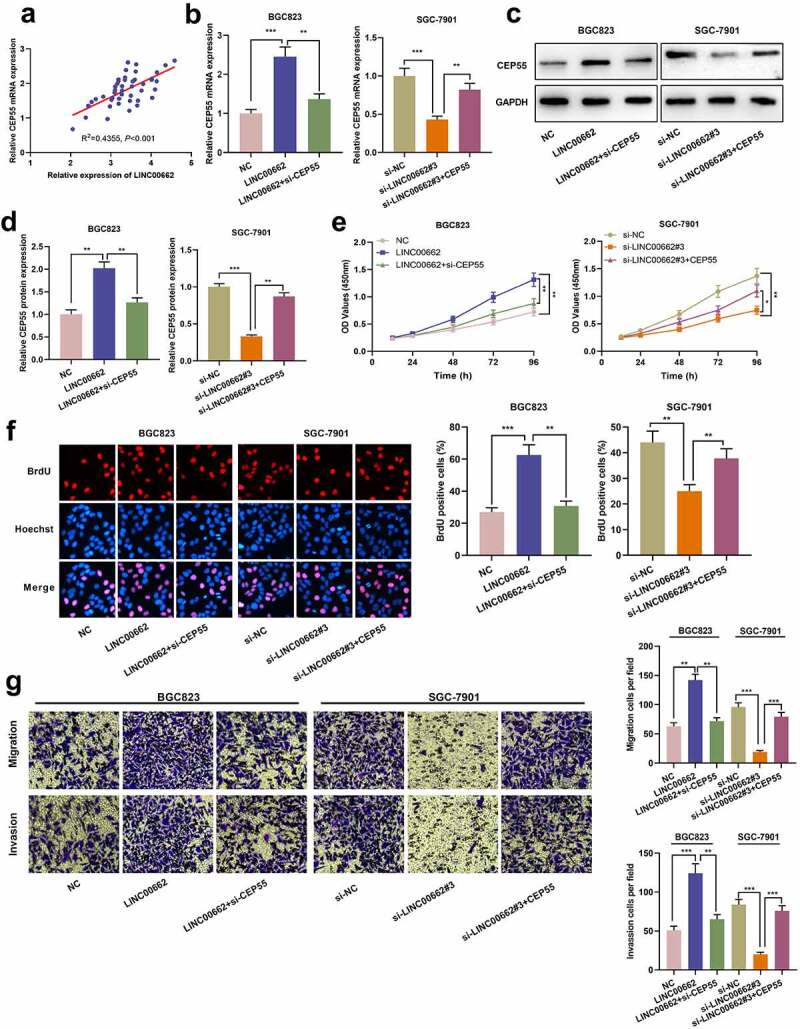
LINC00662 participated in regulating biological behaviors of GC cells by modulating CEP55 expression.

Next, the correlation between CEP55 mRNA expression and LINC00662 expression in GC tissues was analyzed, and the results showed that there was a positive correlation (Figure 5(a)). Subsequently, BGC823 cells were transfected with LINC00662 overexpression plasmid or co-transfected with si-CEP55; SGC-7901 cells were transfected with si-LINC00662#3 or co-transfected with CEP55 overexpression plasmid (Figure 5(b-d)). It was demonstrated that CEP55 knockdown partly abrogated the promoting effects of LINC00662 overexpression on malignant biological behaviors of GC cells whereas overexpression of CEP55 partly counteracted the inhibitory effects of LINC00662 knockdown on these biological behaviors (Figure 5(e-g)). These data suggested that LINC00662 was a participant in regulating GC progression via modulating CEP55.

a. The correlation between LINC00662 and CEP55 mRNA expressions in GC samples (N = 45) was evaluated. b-d. BGC823 cells were transfected with LINC00662 overexpression plasmid or co-transfected with si-CEP55, respectively, and SGC-7901 cells were transfected with si-LINC00662#3 or co-transfected with CEP55 overexpression plasmid. CEP55 mRNA and protein were quantified by qRT-PCR (c) and Western blot (d). e-f. The proliferation of transfected cells was measured employing CCK-8 assay (E) and BrdU assay (F).G. Transwell experiment was conducted to measure the migration and invasion of transfected cells. All of the experiments were performed in triplicate. *P < 0.05, **P < 0.01 and ***P < 0.001.

## Discussion

LncRNA is widely involved in multiple biological processes such as cell proliferation, differentiation, autophagy, apoptosis and so on [23]. Many lncRNAs are aberrantly expressed in human cancers including GC, and they are pivotal regulators in the growth, migration, invasion, apoptosis and drug resistance of GC cells. For instance, lncRNA CERS6-AS1 promotes the progression of GC [24]. What’s more, LINC00240 promotes GC progression via modulating miR-338-5p/METTL3 axis [25]. Additionally, knockdown of LINC00483 represses GC cell viability and induces apoptosis [26]. LINC00662 exhibits a carcinogenic role in multiple types of human cancers. For instance, LINC00662 expedites the proliferation and invasion of liver cancer cells by activating Wnt/β-catenin signaling [10]. Besides, LINC00662, facilitating the metastasis of lung cancer cells through interaction with LIN28, is in association with the unfavorable prognosis of lung cancer patients [11]. We, herein, observed that LINC00662 was remarkably up-regulated in GC tissue samples and cell lines. Furthermore, LINC00662 overexpression promoted the malignant phenotypes of GC cells, but its knockdown exhibited opposite effects. These data suggest that LINC00662 plays a carcinogenic role in GC and was a potential biomarker and therapeutic target for GC.

MiRNAs exert important functions in regulating multiple biological processes [14,27,28]. The roles of a lot of miRNAs in GC have been confirmed in recent years [29–32]. For instance, miR-621 expression in GC exhibits remarkable down-regulation, and it blocks the growth of GC cells by targeting SYF2 [30]. MiR-339-5p represses the malignant phenotypes of GC cells through repressing ALKBH1 [31]. Moreover, reportedly, miR-195-5p blocks GC progression via repressing basic fibroblast growth factors [32]. Besides, accumulating studies prove that lncRNAs, exert pivotal roles in regulating the activity of miRNAs as ceRNAs. For example, SNHG12 participates in regulating IGF1R-induced proliferation and metastasis of osteosarcoma cells by modulating miR-195-5p [33]; BANCR regulates Wnt/β-Catenin signaling pathway by sponging miR-195-5p, thereupon promoting pancreatic cancer progression [34]. In the current research, LINC00662 was found to directly target and negatively regulate miR-195-5p. Additionally, transfecting miR-195-5p mimics partially abolished the promotion of biological behaviors triggered by LINC00662 overexpression, indicating that LINC00662, via modulating miR-195-5p, promotes GC progression.

CEP55 (also known as FLJ10540 or C10orf3), as a component of the centrosome of cells, is recognized as a regulator of cell cycle and mitosis [18]. CEP55 participates in spindle formation by anchoring microtubule polymerization associated proteins, thereupon regulating cell proliferation [18]. Except in thymus and testis, CEP55 is weakly expressed in other mature tissues [35]. Overexpression of CEP55 can trigger the dysfunction of mitosis, giving rise to the presence of multinucleated cells [36]. CEP55 is in close relationship with the occurrence and development of human cancers [19–21]. For instance, CEP55, highly expressed in osteosarcoma, facilitates the proliferation and invasion of tumor cells by activating AKT signaling [37]. In pancreatic cancer, it is in association with the adverse prognosis of the patients; its overexpression promotes the aggressiveness of cancer cells via activating NF-κB signaling [38]. Importantly, it is reported that knockdown of CEP55 suppresses GC cell proliferation by inducing G2/M cell cycle arrest [21]. Herein, it was revealed that, CEP55 was negatively regulated by miR-195-5p. What’s more, knockdown of CEP55 counteracted the effects of LINC00662 overexpression on GC biological behaviors, indicating that LINC00662 participated in GC progression by regulating miR-195-5p/CEP55 axis.

## Conclusion

Our work authenticates that LINC00662 is capable of regulating CEP55 by targeting miR-195-5p, thereupon facilitating the malignant biological behaviors of GC cells. Our work presents novel insights into the diagnosis and therapy of GC.

## Supplementary Material

Supplemental Material

## Data Availability

The data used to support the findings of this study are available from the corresponding author upon request.

## Research highlights

1. LINC00662 is up-regulated in gastric cancer tissues, and it promotes the malignant phenotypes of gastric cancer cells.
2. LINC00662 can repress the expression of miR-195-5p in gastric cancer cells.
3. LINC0062 positively regulates the expression of CEP55 in gastric cancer cells via repressing miR-195-5p.
