# Supplementary figures and images for: Long intergenic non-protein coding RNA 662 accelerates the progression of gastric cancer through up-regulating centrosomal protein 55 by sponging microRNA-195-5p
Source: Bioengineered. 2022 Jan 17;13(2):3007–18. doi: 10.1080/21655979.2021.2023978 (PMC8974125; doi:10.1080/21655979.2021.2023978)

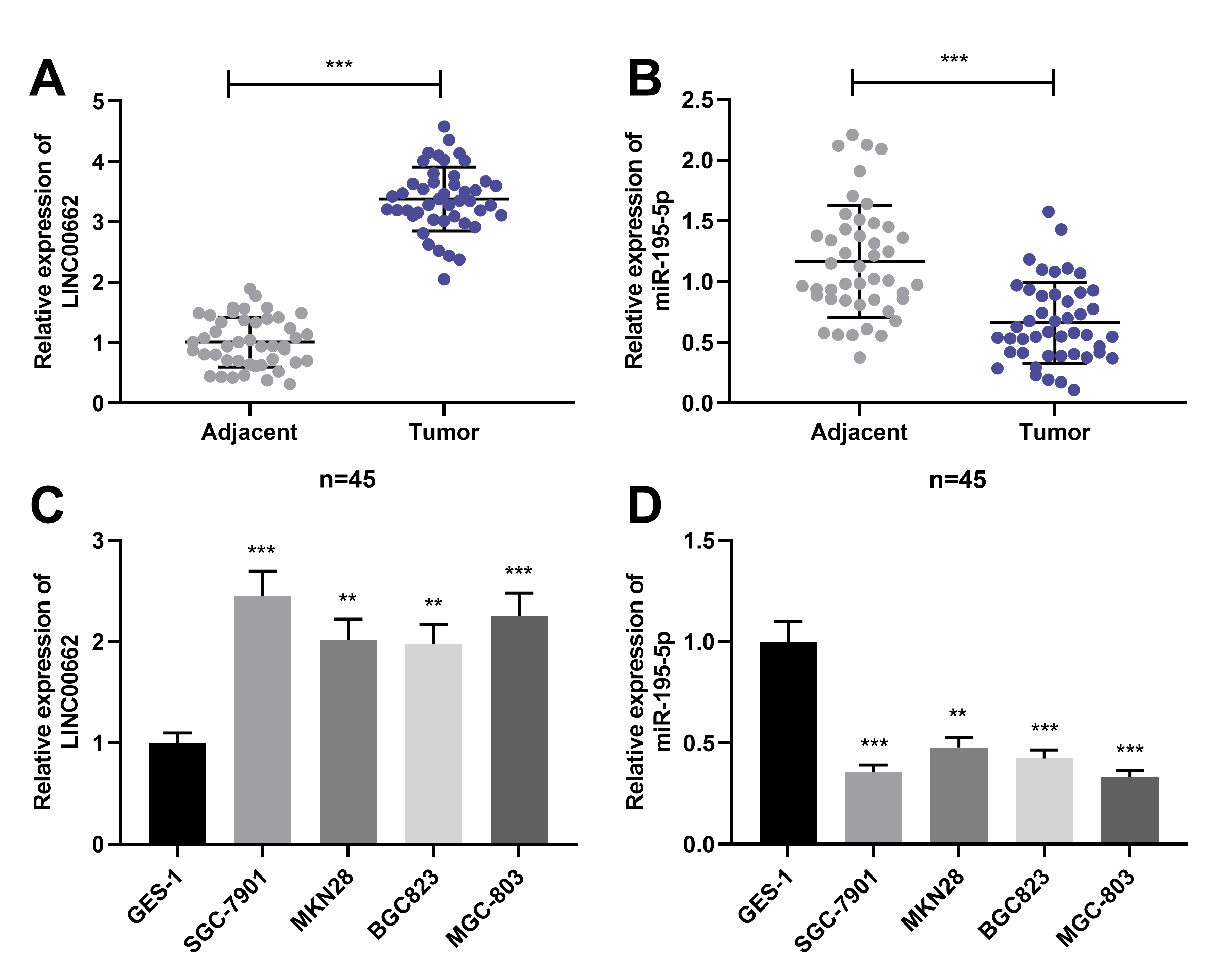

Supplement: Supplemental Material [file KBIE_A_2023978_SM3428.zip › supplementary/Supplementary Figure 1.tif]
